# Drug-Induced Lupus Secondary to Ethosuximide in Association with Acute Tubulointerstitial Nephritis and Nephrotic Syndrome

**DOI:** 10.3390/pediatric14020026

**Published:** 2022-04-14

**Authors:** Rasha Aly, Xu Zeng, Kiran Upadhyay

**Affiliations:** 1 Division of Pediatric Nephrology, Department of Pediatrics, University of Florida, Gainesville, FL 32608, USA; rashaaly@ufl.edu; 2 Division of Anatomic Pathology, Department of Pathology, University of Florida, Gainesville, FL 32608, USA; xu.zeng@ufl.edu

**Keywords:** drug-induced lupus, ethosuximide, tubulointerstitial nephritis, nephrotic syndrome

## Abstract

**Background**. Drug-induced lupus (DIL) is an autoimmune phenomenon where the patient develops lupus-like symptoms after exposure to a long-term medication. **Case Summary**. Here we describe a 10-year-old female with absence seizures who developed a lupus-like syndrome after being on ethosuximide for three months. She presented with nephrotic syndrome (NS) and acute kidney injury. Four weeks prior to presentation, she had been prescribed a seven-day course of oral amoxicillin for submental swelling after dental extraction. Investigations showed high titer of antinuclear antibody (ANA) and anti-double stranded DNA, elevated serum IgE level, and positive Coombs’ test, along with positive anti-histone antibodies. Renal biopsy showed features of acute tubulointerstitial nephritis (TIN) and partial podocyte foot process effacement without evidence of lupus nephritis. The patient had an excellent response to the steroid therapy with remission within two weeks. The patient remained in remission for two months as evaluated during the most recent follow-up; the autoimmune antibodies and immunoglobulin E trended down. Ethosuximide has been reported to cause DIL, however its possible association with TIN has not been reported. Although amoxicillin could have caused the TIN and NS in this patient, a possible novel association of ethosuximide with this nephrotic-nephritic presentation (NNP) cannot be ruled out. **Conclusions**. A renal histology is important to determine the accurate etiology of NNP in patients with DIL. Further studies are necessary to determine any possible causal effect of ethosuximide with NNP.

## 1. Introduction

Drug-induced lupus (DIL) is an autoimmune phenomenon where the patient develops lupus-like symptoms after exposure to certain medications, usually months to years after initiation [1]. It has been found to be more common in the female gender and Caucasian race [1]. DIL is considered as an example of an environmental trigger leading to activation of autoimmunity resulting in development of lupus-like symptoms in a genetically susceptible individual [1]. The drugs are divided into therapeutic classes and their approximate risk levels are based on the number of case reports. Hydralazine was one of the first agents to be associated with the development of lupus-like symptoms [2]. DIL differs from a classic drug hypersensitivity reaction as symptoms do not present rapidly upon challenge given that it is not associated with the memory of prior exposure [3]. Furthermore, there is a strong correlation between the development of DIL and the accumulative dose; lupus-like symptoms develop after exposure to the medication for months or years [3,4].

Here we report a case of DIL associated with ethosuximide in a patient who presented with acute kidney injury (AKI) and nephrotic syndrome (NS). The kidney biopsy revealed acute tubulointerstitial nephritis (TIN) and podocyte injury without evidence of lupus nephritis. As drug-induced TIN represents two-thirds of total TIN cases with antibiotics responsible for approximately 30–50% of these cases [3], amoxicillin usage in our patient could have contributed to the development of AKI and NS. However, the simultaneous presentation of DIL, which has not been described with amoxicillin, was an interesting association. Future studies need to look at the potential risk of AKI and NS with ethosuximide usage.

## 2. Material and Methods

This is a retrospective case study, and the family of the patient provided the consent for the study.

## 3. Case Presentation

A 10-year-old African American female presented with bilateral lower extremity edema, abdominal distension, malaise, loss of appetite, nausea, and weight gain for two weeks. There was no history of fever, gross blood in urine or joint pains. Past medical history was significant for absence seizures, and she was started on ethosuximide 250 mg daily three months prior to presentation. The staring spells decreased from 8–10 episodes per day to 5–6 episodes per week after initiation of ethosuximide. Four weeks prior to presentation, she developed submental swelling following a dental extraction and received a seven-day course of amoxicillin. The patient had no history of prior allergic reactions with amoxicillin. There was no history of recent usage of non-steroidal anti-inflammatory drugs (NSAID), over the counter medications or proton pump inhibitors. There was no recent viral upper respiratory or gastrointestinal illness, streptococcal throat or skin infection and skin rash. Family history was not suggestive of systemic lupus, NS, other autoimmune diseases, renal failure, dialysis, kidney transplantation, or sickle cell disease. Her immunization was up to date and she had normal growth and development.

On examination, the vital signs showed an afebrile child with a heart rate of 74 beats per minute, respiratory rate 20 per minute, blood pressure 122/86 mm Hg (>95th percentile for age, gender, and height), and oxygen saturation of 96% on room air. Her current weight was 49.4 kg (95th percentile) and the baseline weight prior to presentation was 41 kg. Physical examination showed puffiness of eyes and face, abdominal distension, and 1+ bilateral pitting edema of the lower extremities. There were no oral ulcers, joint swelling, tenderness or erythema. Skin examination showed some non-itchy and non-painful excoriated pustules on the frontal hairline and upper thighs posteriorly. There was no malar or discoid rash. Eye examination showed no redness; a formal ophthalmology examination showed no uveitis. Mental status was normal without seizures.

Initial renal function test and complete blood count values are shown in Table 1. Prior renal function test was unknown. Differential blood count showed no eosinophilia. Liver enzymes were normal. Serum creatinine kinase was normal. Urinalysis showed specific gravity ≥1.030, pH 6.0, ≥500 protein, one red cell per high power field, two white cells per high power field, negative nitrite, and leukocytes. Urine eosinophil was not obtained. Random urine protein to creatinine ratio was 5.4 mg/mg (normal < 0.2 mg/mg). Urine culture was negative. Subsequent blood count showed drop in hemoglobin to 10.2 gm/dL with stable white cell count and platelet count. There was Coombs’ positive autoimmune hemolytic anemia. Anti-nuclear antibody (ANA), anti-double-stranded (ds) DNA antibody and anti-histone antibodies (8.5 units, normal range: 0–1 unit, 1–1.5: weak positive, 1.6–2.5: moderate positive, 2.6 units or greater: strong positive) were all strongly positive (Table 2). Anti-smith antibody was negative (5 AU/mL, normal range: 0–40 AU/mL). Ribonucleic protein, SCL-70 antibody, SS-A and SS-B were all negative. Serum immunoglobulin E (IgE) was elevated at 5811 KU/L (normal ≤ 696 KU/L) but there was normal serum IgG and IgA. Nasopharyngeal SARS-CoV-2 polymerase chain reaction (PCR) was negative. Varicella zoster DNA PCR and Epstein–Barr virus DNA PCR were negative.

**Figure 1:**
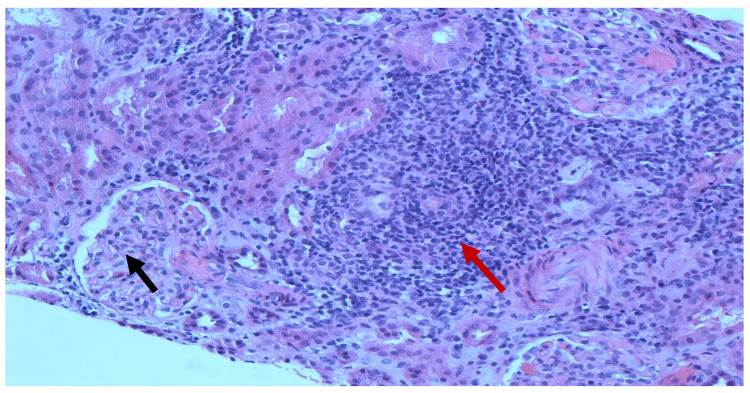
Renal biopsy. Light microscopy (H&E) showing mild increase in mesangial cellularity with moderate increase in the mesangial matrix (black arrow) along with moderate to severe interstitial inflammation, comprising lymphocytes/mononuclear cells, plasma cells, some eosinophils, and rare neutrophils (red arrow).

**Figure 2:**
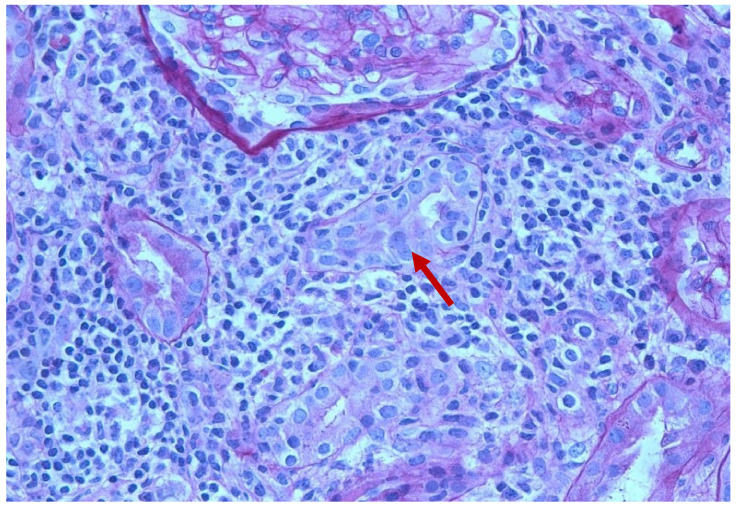
Renal biopsy. Light microscopy (H&E) showing tubulitis (red arrow) with severe interstitial inflammation.

**Figure 3:**
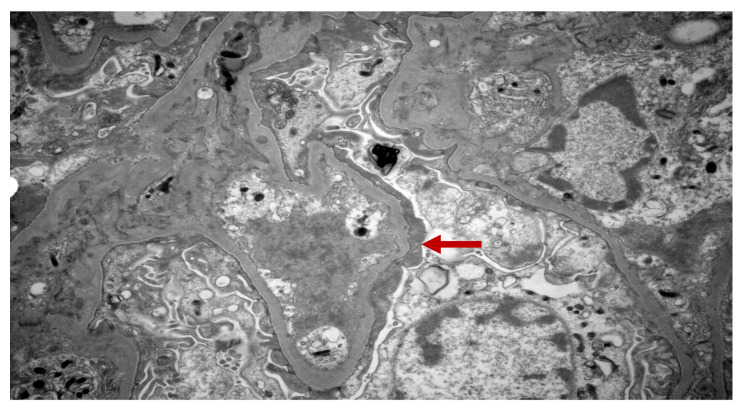
Renal biopsy. Electron microscopy showing absence of immune deposits or tubulo-reticular inclusions but presence of partial podocyte foot processes effacement (red arrow).

Chest X-ray revealed no pleural effusion, pneumothorax, consolidation, or acute abnormalities. Renal sonogram demonstrated bilateral echogenic kidneys with right kidney measuring 11 cm in length and left kidney measuring 11.2 cm in length without hydronephrosis. A percutaneous sonogram-guided renal biopsy which was obtained three days after the onset of current signs and symptoms showed two portions of renal cortical tissue with ten glomeruli. Light microscopy showed a segmental mild increase in mesangial cellularity with moderate increase in the mesangial matrix. There was no segmental sclerosis, cellular crescent, nor area of segmental necrosis (Figure 1). The tubular atrophy and interstitial fibrosis were mild. There was moderate to severe interstitial inflammation, comprised of lymphocytes/mononuclear cells, plasma cells, eosinophils, and rare neutrophils, associated with severe tubulitis and tubular damage (Figure 2). No granulomas were seen. There was no active arteritis. The immunofluorescence study was negative for IgA, IgG, IgM, C3, C1q, kappa, and lambda light chains. Electron microscopy showed a normal glomerular basement membrane without immune deposits nor tubular-reticular inclusions. There was partial podocyte foot processes effacement (Figure 3). These findings were most consistent with acute TIN with podocyte injury.

The patient was treated with albumin/diuretic infusions for the symptomatic relief of edema and the weight trended down to near her baseline at discharge. Due to severe TIN and NS, a course of steroids, prednisone 60 mg daily, was started with gradual tapering over six weeks. Serum creatinine trended down to 1.13 mg/dL at discharge. Due to concerns of DIL, ethosuximide was changed to zonisamide after consultation with neurology and switched again after three weeks to sodium valproate for better seizure control. Rheumatology consultation was carried out, who agreed with the diagnosis of ethosuximide-induced DIL. Mild anemia persisted with serum hemoglobin of 10.8 gm/dL at the time of discharge.

The edema resolved within two weeks and her weight returned to the baseline. Hypertension, which was thought to be secondary to fluid overload and steroid usage, was managed with amlodipine. Blood pressure normalized and hence amlodipine was discontinued after completion of steroid therapy. At two weeks follow-up, the serum creatinine was 0.6 mg/dL (schwartz eGFR 90 mL/min/1.73 m^2^), serum albumin improved to 3.4 g/dL from 2.2 g/dL at discharge, urinalysis was negative for blood and protein, with normal spot urine protein creatinine ratio of 0.12 mg/mg. Serum 25 hydroxy-vitamin D and IgE improved. At two months follow-up, ANA was weakly positive with titer of 1:160 (homogenous pattern), dsDNA antibody was 10 IU (normal range 0–24 IU, IgG ELISA, ARUP laboratories, Salt Lake City, UT, USA), and anti-histone antibody was 1.0 unit (Table 1).

## 4. Discussion

DIL is considered to be a type B (hypersensitivity) drug reaction which can be further sub-classified into type I-IV hypersensitivity reaction. The clinical picture of DIL is mainly heterogeneous and generally characterized by systemic symptoms such as fever, weight loss, anorexia, and arthralgia. Arthritis and serositis may occur (particularly in procainamide-induced cases), whereas central nervous system, renal, gastrointestinal, and hematologic abnormalities and cutaneous lesions are rare [4,5]. Organ-limited forms, particularly subacute cutaneous lupus and discoid lupus, have also been reported [6]. Glomerulonephritis associated with DIL is rare but has been reported with hydralazine, sulfasalazine, propylthiouracil, penicillamine and anti-TNFα therapy [7]. Laboratory findings typically show a positive ANA in a homogenous pattern as the autoantibodies target the nuclear histone proteins. Although anti-histone antibodies are the hallmark of DIL and are positive in up to 95% of cases, anti-dS DNA antibodies have also been observed, particularly in TNFα inhibitor induced DIL [8]. The strong presence of anti-histone antibodies and the rarity of anti-ds DNA antibodies is in strong contrast to idiopathic SLE [1,9]. Anemia is found in <45%, positive Coombs’ test in <30%, anti-smith antibodies in <5% and low serum complement level is rare [4,5]. DIL usually resolves without intervention after removal of the offending agent [10]. However, it may take months for the autoimmune labs to normalize.

Some of the drugs known to cause DIL are isoniazid, procainamide, hydralazine, TNF α inhibitors (such as etanercept, infliximab and adalimumab), and minocycline, among others [1,2]. Incidence of DIL with drugs such as procainamide and hydralazine is higher with rates about 20–30% and 5–10%, respectively, during the first year of therapy [1,11]. With regards to anticonvulsants, carbamazepine, phenytoin, primidone, and ethosuximide all have been shown to have a very low risk of inducing DIL [1,6]. While DIL tends to be less severe than SLE, the diagnosis can be challenging especially with the medications not commonly known to cause DIL, such as ethosuximide. The incidence of DIL with anti-TNFα therapy is about 0.1% [12].

The diagnosis of DIL is established based upon the history of sufficient and continued exposure to a specific drug, at least one symptom compatible with systemic lupus, no prior history of lupus and resolution of symptoms within weeks after discontinuation of the offending agent [5]. Our patient in this report met all the criteria for DIL.

The proposed mechanisms of autoantibody induction by the lupus-inducing drugs include an immune response to the drug in the form of a hapten or to a self-antigen altered by the drug which then induces antibodies that cross-react with or cause spreading of the immune response to the native self-macromolecules. Other mechanisms include direct activation of B and/or T lymphocytes by the drug, drug-specific T cells activating autoreactive B cells presenting the drug, and prevention of establishment of immune self-tolerance by the drug [1]. The reactive metabolites of the drug generated by oxidation of drugs by activated neutrophils and not the drug itself are perhaps the likely cause of initiation of autoimmunity given the time lag between the initial exposure and the appearance of serologic abnormalities [1]. Those with genetic susceptibility, such as individuals who are slow acetylators with genetic deficiency of N-acetyltransferase, are at higher risk of developing DIL as compared to the fast acetylators, especially with drugs that are predominantly metabolized by acetylation such as procainamide and hydralazine [13]. Some studies suggest an association between certain human leukocyte antigen (HLA) types (HLA-DR2, HLA-DR3) and increased risk of autoimmunity and DIL; however, these findings are not always consistent [6,14]. In addition, patients with inherited complement C2 or C4 deficiency have been associated with SLE or related to drug effects [15]. Our patient had normal serum complements. Some studies also reported that certain drugs, such as hydralazine, penicillamine, isoniazid, and metabolic products of procainamide, could inhibit the covalent binding of complement C4 that subsequently could inhibit the activation of complement C3 in the classical complement pathway, resulting in preventing the clearance of immune complexes and increasing the chance of the autoimmunity [16,17]. Furthermore, drugs with a larger molecular weight, such as procainamide, are oxidized by activated neutrophils resulting in the production of a toxic metabolite called procainamide hydroxylamine (PAHA). PAHA, together with myeloperoxidase and reactive oxygen species released during the oxidative metabolism of procainamide, may contribute to cytotoxicity [18,19,20]. The small molecules can bind to proteins and act as a hapten, which then stimulate immune responses [21,22]. With regards to ethosuximide, the pathophysiology of DIL could be related to the host genetic risk factor of being a slow acetylator or inherited complement deficiency. Acetylation was not checked in our patient and her serum complements C3 and C4 were normal. Furthermore, ethosuximide has a small molecular weight that may act as a hapten.

Over two-thirds of TIN cases are drug-induced [3]. Antibiotics, NSAIDs, and proton pump inhibitors are the most frequent drugs associated with drug-induced TIN [3]. Drug-induced TIN typically occurs 10–20 days after initiation of the drug; however, this period can be as short as one day after some antibiotics or as long as several months with NSAIDs [23,24]. It can also occur de novo in response to a medication that was previously tolerated by the patient, as could have occurred in our case [25]. Nephrotic proteinuria and AKI have been typically associated with the usage of NSAID; in our patient, there was no history of prior exposure to NSAID. Rash can develop in up to 50% and eosinophilia is variable. However, absence of eosinophilia and eosinophiluria is of limited value in excluding the diagnosis of TIN [26]. Antibiotics are responsible for approximately 30–50% of these cases [27]. A review of the published literature shows that there are reports of amoxicillin-induced TIN in the children supporting our case with the history of seven-day course of amoxicillin following tooth extraction four weeks prior to the presentation [28,29,30]. The development of antibiotic-induced TIN is not dose-dependent, and a recurrence or exacerbation of TIN can occur with a second exposure to the same or a related drug [31].

Drug-induced TIN is thought to be a T-cell mediated type-IV hypersensitivity reaction due to the latent period between drug exposure and the development of a rash and eosinophilia. Furthermore, the drugs may act as haptens that bind to the cytoplasmic or extracellular components of tubular cells during secretion to modify the endogenous response to native renal proteins or induce an autoimmune reaction to the tubular basement membrane through molecular mimicry [3]. In some patients’ serum, IgE level is elevated as in our patient, suggesting a type 1 hypersensitivity reaction [32]. As early as seven days after drug exposure, the early inflammatory lesions in TIN can begin to evolve into irreversible interstitial fibrosis. This likely explains why, even with the best available management, only 40–50% of patients with TIN experience complete renal recovery. Hence, rapid identification and withdrawal of the suspected drug along with early treatment with corticosteroids usually within the first five days can reduce the amount of tubulointerstitial fibrosis that may develop leading to incomplete recovery of renal function [27]. A generally accepted consensus is that in those patients with suboptimal (or absence of) response to the discontinuation of drugs, or in those with severe TIN or worsening AKI, early therapy with steroids is appropriate [25].

Ethosuximide has not been described in the literature as a causative agent of drug-induced TIN, but in our case, we could not exclude its contribution to NS and TIN given the concurrent presence of DIL. Korinthenberg et al. performed a cross-sectional study of 59 patients without prior renal history and who had been on anti-epileptic monotherapy including carbamazepine, valproic acid, phenytoin, and ethosuximide for at least three months [33]. The proximal tubular function was measured by the urinary excretion of α1 microglobulin and of the tubular enzymes N-acetyl-beta-D-glucosaminidase, alanine-amino-peptidase, and fructose-l, 6-di-phosphatase. The distal tubular function was examined by the 24-hr excretion of Tamm–Horsfall protein. On treatment with carbamazepine (*n* = 27) and phenytoin (*n* = 8), the excretion of α1 microglobulin was significantly increased, as compared with the healthy controls. On valproate (*n* = 20), ethosuximide (*n* = 9), and phenytoin (*n* = 8), the excretion of N-acetyl-beta-D-glucosaminidase was significantly increased. This study showed that these anticonvulsants may cause functional disturbance of the proximal tubules. Anticonvulsants other than ethosuximide have been reported to be associated with TIN. Yoshikawa et al. reported a 14-year-old child with TIN associated with sodium valproate [34]. Eijgenraam et al. reported carbamazepine-associated acute TIN [35]. Matta et al. described a 27-year-old patient with lamotrigine-induced acute interstitial nephritis [36]. Other antiepileptics that have been described to cause TIN are phenytoin and phenobarbital [37].

Drug-induced nephrotoxicity can manifest more commonly as tubulointerstitial injury and less commonly as glomerular injury [38]. Drug-induced glomerular disease can be due to either direct cellular injury or via immune-mediated injury [7]. The three major cellular targets in glomerular injury are the podocytes, endothelial cells, and mesangial cells [39]. In addition to the direct cellular toxicity, some of the drugs can also elicit an immune response resulting in the generation of autoantibodies and clinical autoimmune disease such as immune complex or pauci-immune GN [7]. Drug-induced ANCA vasculitis, drug-induced lupus and drug-induced membranous nephropathy have all been described [7]. Drugs such as NSAIDs, interferons, bisphosphonates, ranitidine, rifampin, sirolimus, anabolic steroids, and chronic lithium use are known to cause podocytopathy manifesting either as minimal change nephrotic syndrome or focal segmental glomerulosclerosis lesions [40,41,42,43]. The typical biopsy lesion in NSAID-associated TIN shows absence of glomerular lesions with fusion of the podocyte foot processes; however, in our case, there was mild increase in glomerular mesangial cellularity with partial loss of podocyte foot processes. Pamidronate has been shown to impair podocyte energetics, disruption of the cytoskeleton and altered cell signaling [44]. Whether there is any role of ethosuximide and other anticonvulsants in inducing podocyte injury is unknown and hence needs to be studied further.

## 5. Limitations

Limitations of our report include absence of testing for urine eosinophils and slow acetylation. Given the usage of amoxicillin four weeks prior to the presentation of TIN, NS and DIL, the definite cause-and-effect relationship between ethosuximide and the clinical presentation could not be established. However, given that there are no reports of DIL caused by amoxicillin, it is possible that the clinical presentation was induced by two agents: ethosuximide causing DIL and amoxicillin causing TIN and NS or ethosuximide causing all three manifestations, i.e., DIL, TIN and NS. This will need to be looked at in larger further studies and remains a mere speculation at this time. A long-term follow-up would also be helpful to evaluate the trend of the autoantibodies.

## 6. Conclusions

The case report presents a rare association of DIL with ethosuximide along with its possible role in induction of TIN and NS in children. This report highlights the importance of renal histology to determine the accurate etiology of NS and AKI in patients with lupus-like symptoms. For better understanding of the clinical spectrum of anticonvulsant-induced nephrotoxicity, larger studies are needed to confirm the cause-and-effect relationship.

## Figures and Tables

**Table 1:** Laboratory values. ANCA, anti-neutrophil cytoplasmic antibody; ASO, anti-streptolysin O; BUN, blood urea nitrogen; eGFR, estimated glomerular filtration rate.

	Initial Labs
Sodium	134 mmol/L
Potassium	4.1 mmol/L
Chloride	102 mmol/L
Bicarbonate	24 mmol/L
BUN	41 mg/dL
Creatinine	1.5 mg/dL (schwartz eGFR 40 mL/min/1.73 m^2^)
Albumin	1.7 g/dL
White blood cell count	7.6 × 10^9^/L
Hemoglobin	11.7 gm/dL
Platelet count	443 × 10^9^/L
ANCA	Negative
ASO	Negative
C-reactive protein	Normal
Uric acid	3.7 mg/dL

**Table 2:** Laboratory values. ANA, antinuclear antibody; Ab, antibody; ds, DNA, deoxyribonucleic acid; double-stranded; ELISA, enzyme-linked immunosorbent assay; ENA, extractable nuclear antigen; IFA, immunofluorescence assay; Ig, immunoglobulin.

	Initial Labs	2 Months Follow-Up
ANA pattern	Homogeneous	Homogeneous
ANA Titer	1:1280	1:160
DNA Ab (dS) Crithidia, IFA	1:2560	1:80
ds DNA Ab, IgG ELISA, IU	287	10
Histone Ab, U	8.5	1
Myeloperoxidase Ab, AU/mL	7	
Proteinase 3 Ab, AU/mL	7	
Ribonucleic Protein Ab, ENA IgG	6	
Scleroderma (SCL-70) Ab, AU/mL	10	
SSA (Ro) IgG Ab, AU/mL	5	
SSB (La) (ENA) Ab IgG, AU/mL	3	
Smith Ab, IgG, AU/mL	5	
25 hydroxy vitamin D, ng/mL	<7.0	9.39
Albumin, gm/dL	2.2	3.4
C3 Complement, mg/dL	94	104
C4 Complement, mg/dL	13	15
IgG, KU/L	1227	
IgM, KU/L	139	
IgA, KU/L	49	
IgE, KU/L	5811	3451

## Data Availability

The data presented in this study are available on request from the corresponding author.
